# Targeted therapy in the treatment of lung cancer in Iceland 2010–2023

**DOI:** 10.2340/ao.v65.45118

**Published:** 2026-04-09

**Authors:** Stefanía Ásta Tryggvadottir, Guðlaugur V. Stefansson, Helgi Birgisson, Örvar Gunnarsson, Tómas Guðbjartsson, Hronn Harðardóttir, Bylgja Hilmarsdottir, Rósa B. Barkardóttir, Sigurdis Haraldsdottir

**Affiliations:** a Faculty of Medicine, University of Iceland, Reykjavik, Iceland; b Icelandic Cancer Registry, Reykjavik, Iceland; c Department of Oncology, Landspitali – The National University Hospital of Iceland, Reykjavik, Iceland; d Molecular Pathology Unit, Landspitali - The National University Hospital of Iceland, Reykjavik, Iceland; e Department of Pathology, Landspitali - The National University Hospital of Iceland, Reykjavik, Iceland; f University of Iceland; BioMedical Center (BMC), Reykjavik, Iceland

**Keywords:** Nonsmall cell lung cancer, next-generation sequencing, precision medicine, epidermal growth factor receptor (EGFR), real-world data

## Abstract

**Background and purpose:**

Lung cancer is the third most common malignancy in Iceland and remains the leading cause of cancer-related mortality. Lung cancer may harbor driver mutations affecting the function of *Epidermal growth factor receptor (EGFR), Anaplastic lymphoma kinase (ALK), ROS proto-oncogene 1 (ROS1), B-raf proto-oncogene (BRAF), RET proto-oncogene (RET), MET proto-oncogene (MET),* and *NTRK*, which can influence the selection of targeted therapies and treatment outcomes. In Iceland, *EGFR* testing was initiated in 2005, and in 2016, multigene targeted panel became standard for tumor testing. The objective was to determine the uptake of testing and frequency of targeted mutations in lung cancer nationwide, the utilization of targeted therapies, and duration of such treatments.

**Patients/material and methods:**

Data on lung cancer diagnoses and stage at diagnosis were obtained from the Icelandic Cancer Registry, and molecular testing results were retrieved from the Department of Pathology at Landspitali University Hospital. Treatment data and outcomes were obtained from a central prescription/death registry and chart reviews.

**Results:**

From 2010 to 2023, 2,528 patients were diagnosed with lung cancer, and 25% underwent molecular tumor testing, with 90% of stage IV adenocarcinomas tested in 2023. During comprehensive molecular testing in 2016–2023, targeted mutations were detected in 19.3% of tested patients: *EGFR* 9.9%, *BRAF* 2.3%, *MET* 2.7%, *HER2* 1.2%, *ALK* 2.7%, *ROS1* 0.6%, and *RET* 0.2%. Among patients found to have targeted mutations (2010–2023), 61.2% received targeted therapy; 33.8% remained on therapy for ≥ 12 months, and 13.5% for ≥ 24 months.

**Interpretation:**

The use of molecular testing has increased significantly in the last 20 years, and the adaptation of new targeted therapies has been rapid.

## Introduction/Background

Lung cancer remains the leading cause of cancer-related death worldwide and in Iceland [1]. Although the incidence of lung cancer has declined in recent decades due to reduced smoking rates, it continues to represent a major public health burden. In Iceland, lung cancer is the second most common cancer among females and the fourth most common among males, with an average age at diagnosis of 68 years. Smoking rates have decreased significantly in Iceland in the last 30–40 years; in 1991, 31.2% of people in Iceland aged 18–89 smoked daily, compared to 5.7% in 2023 [2, 3].

Over the past two decades, major advances in the molecular understanding of non–small cell lung cancer (NSCLC) have led to the introduction of targeted therapies directed against specific driver mutations such as *EGFR*, B-raf proto-oncogene (*BRAF)*, erb-b2 receptor tyrosine kinase (*HER2), KRAS*, MET proto-oncogene. (*MET)*, and RET proto-oncogene. (*RET)* [4]. Adenocarcinomas can harbor oncogenic driver mutations that are more commonly seen in females and nonsmokers, whereas squamous cell carcinomas are more strongly associated with smoking and have fewer targetable mutations [5]. EGFR inhibitors were the first targeted therapy, introduced in the early 2000s [6].

At Landspitali University Hospital, molecular testing of lung cancer samples was first implemented in 2005 using Sanger sequencing on selected *EGFR* exons (see Supplementary Figure 1). In 2014, this method was replaced by Quantitative Polymerase Chain Reaction (qPCR), and by 2016, the TruSight Tumor 15 panel enabled the detection of mutations in 15 cancer-related genes, including *EGFR*, *BRAF*, *HER2, KRAS*, *MET*, *PIK3CA*, and *RET* [7]. Staining for ALK and ROS1 was also implemented in 2016. The TruSight Oncology 500 panel was introduced in 2019, detecting mutations in 523 genes, as well as assessment of microsatellite instability and tumor mutational burden [8]. In 2021, the Archer LungFusionPlex panel was implemented to identify gene fusions across 14 genes [9], and if ALK or ROS1 staining was positive, the samples underwent FISH testing. Since 2023, the VariantPlex Expanded Solid Tumor panel has been used, identifying a wide range of genomic alterations across 76 genes [10].

This rapid evolution of molecular tests has been a key driver of therapeutic advances in NSCLC, improving outcomes not only for patients with advanced-stage disease but also for those with limited-stage tumors [4]. Molecular testing has therefore become an essential part of NSCLC management, guiding treatment decisions and improving survival. The implementation and evolution of molecular testing and targeted therapy in NSCLC in Iceland have not been described before, and it is important to have high coverage of molecular testing for patients. EGFR testing has now been performed for two decades and multigene panel testing for almost a decade, and as all testing is centralized, real-world data analysis are easily accessible.

The aim of this study was to determine the frequency of targeted driver mutations and gene rearrangements in lung adenocarcinoma samples in Iceland from 2010 to 2023, to assess the proportion of patients who received targeted therapy, and to evaluate the duration of these treatments.

## Patients/materials and methods

This was a retrospective, population-based cohort study with approval from the central institutional review board (VSN 22-016). All patients diagnosed with NSCLC in Iceland between January 1, 2010 and December 31, 2023 were identified through the Icelandic Cancer Registry**,** which contains comprehensive data on all cancer diagnoses with > 99% accuracy [11]. Information on disease stage at diagnosis was available for cases diagnosed from 2019 to 2023.

**Figure 1:**
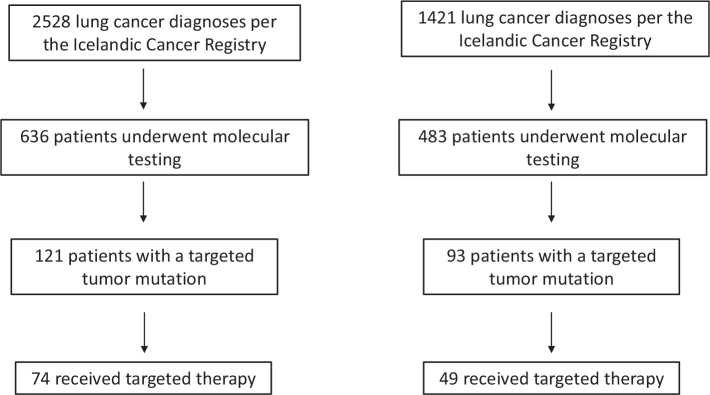
Flowchart for patient selection. Left panel showing testing in 2010–2023, EGFR testing (2010–2023) and multigene panel sequencing (2016–2023). Right panel showing testing in 2016–2023 when multigene panel sequencing was performed.

Results of molecular testing performed between 2010 and 2023 were retrieved from the Department of Pathology, Landspitali University Hospital, where all molecular analyses for lung cancer in Iceland are centralized (see Figure 1). *EGFR* mutation results were available for the whole period, and from 2016, multiple genes were analyzed for mutations after implementation of multigene next-generation sequencing (NGS) panels; in 2016, the TruSight Tumor 15 (TST15) panel was implemented; in 2019, the TruSight Oncology 500 (TSO500, Illumina, implemented for clinical samples in collaboration with deCODE genetics); in 2021, the ArcherFUSIONPlexLung (IDT); and in 2023, the ArcherVARIANTPlexExpanded Solid Tumor (IDT). With these, mutational testing expanded to include additional actionable genes such as *BRAF, HER2, KRAS, MET, RET, ROS1*, and *NTRK*. For this period (2016–2023), the prevalence of various driver mutations was analyzed, including the distribution of *KRAS* subtypes. The ratio of lung cancer samples that underwent molecular testing each year was analyzed for three groups: all lung cancer cases, adenocarcinoma cases, and stage IV adenocarcinoma cases from 2019 to 2023.

Prescription data on targeted therapies were obtained from the Prescription Medicines Registry, while the cause of death was retrieved from the Causes of Death Registry. Medical records from Landspitali (where 80–90% of patients are treated), as well as Sjukrahusid, Akureyri and Heilbrigdisstofnun Sudurlands hospitals, were reviewed to determine the initiation and duration of targeted therapy.

The primary endpoint was the frequency of actionable driver mutations or gene rearrangements in NSCLC in Iceland. Secondary endpoints included the proportion of patients with a targetable mutation who received targeted therapy and the proportion of treated patients who remained on targeted therapy for ≥ 1 and ≥ 2 years. Statistical analyses were performed using R software (version 4.3.2; R Foundation for Statistical Computing, Vienna, Austria). The software was used for data management, descriptive statistics, and graphical visualization.

## Results

### Molecular testing

**Figure 2:**
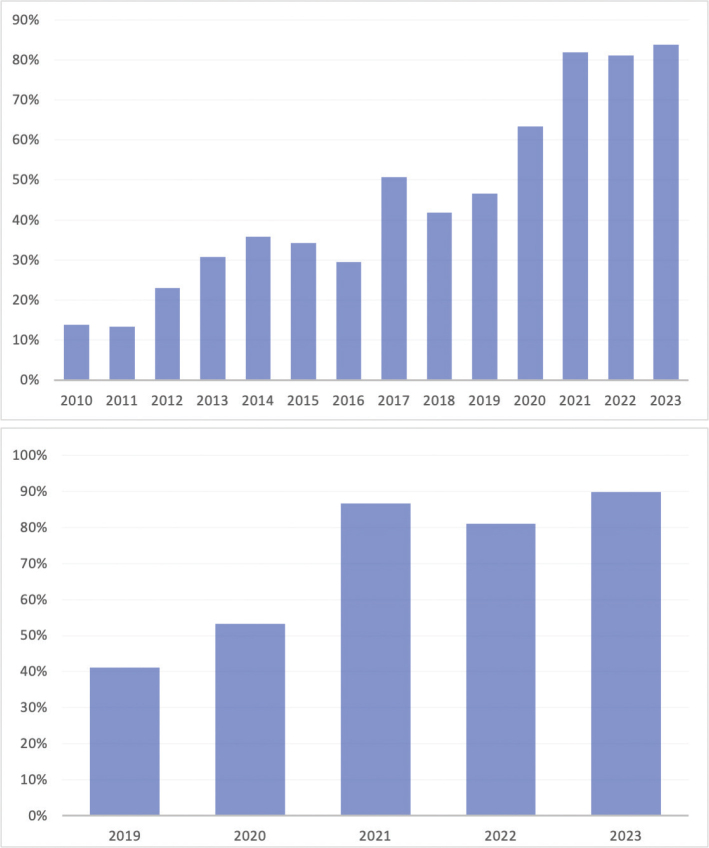
Proportion with molecular testing results examining all adenocarcinoma of the lung (*n* = 1,229) in 2010–2023 (upper panel) and stage IV adenocarcinoma of the lung (*n* = 200) in 2019–2023 (lower panel).

Of 2,528 patients diagnosed with lung cancer in 2010–2023, 636 (25.2%) underwent molecular testing. Patient characteristics are shown in Table 1. The ratio of all lung cancer samples that underwent molecular testing rose from 5.8% in 2010 to 59% in 2023. The ratio of lung adenocarcinoma samples that underwent molecular testing was 13.9% in 2010, 50.7% in 2017, and 83.8% in 2023. Among those diagnosed with stage IV lung adenocarcinoma, 41.2% of lung samples underwent molecular testing in 2019, and in 2023, it had reached up to 89.8% (see Figure 2).

**Table 1:** Patient characteristics in the overall group and comparing patients who underwent molecular tumor testing versus those who did not.

	Tested	Not tested	All
	*n* = 636 (25%)	*n* = 1,892 (75%)	*n* = 2,528
Age, mean (SD), years	68 (±9.8)	71 (±10.6)	70 (±10.4)
Sex			
Females	375 (59%)	999 (53%)	1,374 (54.4%)
Males	261 (41%)	893 (47%)	1,154 (45.6%)
Histology			
Adenocarcinoma	516 (81%)	579 (30.6%)	1,095 (43.3%)
Squamous cell carcinoma	21 (3.3%)	381 (20.1%)	402 (15.9%)
Small cell carcinoma	2 (0.3%)	323 (17.1%)	325 (12.9%)
Other histologies	97 (15.3%)	609 (32.2%)	706 (27.9%)
Diagnosed in			
2010–2014	117 (18.4%)	789 (41.7%)	906 (35.8%)
2015–2019	198 (31.1%)	689 (36.4%)	887 (35.1%)
2020–2023	321 (50.5%)	414 (21.9%)	735 (29.1%)

SD: standard deviation.

### Targeted mutations and therapy

During the study period, the frequency of *EGFR* mutations among lung samples that underwent molecular testing was 11.8% (75/636). The distribution of *EGFR* mutations can be seen in Supplementary Figure 2, with almost half carrying exon 19 deletions. From 2016 to 2023, samples from 483 patients underwent molecular testing, and among those, 19.3% (93/483) had a targetable mutation, where *EGFR* mutations had the highest frequency by far. In *KRAS*, a driver gene frequently mutated in lung cancer and an emerging drug target, the most common mutation was G12Cys. The distribution of mutations in *KRAS* can be seen in Supplementary Figure 2. *KRAS* targeted therapy is not yet standard of care in Iceland.

Overall, there were 121 patient samples that were found to have a targetable mutation during the whole study period, 2010–2023 (see Table 2). Among those, 57% (69/121) had *EGFR* mutations, 14% (17/121) had *ALK* translocation, 2.5% (3/121) had *ROS1* translocation, 9.9% (12/121) had *BRAF* V600E mutation, 5.8% (7/121) had *HER2* mutation, 0.8% (1/121) had *RET* mutation, and 11.6% (14/121) had *MET* translocation. No *NTRK* fusions were found. Among those 121 patients, 61.2% (74/121) were treated with targeted therapy; all treated patients had advanced disease.

**Table 2:** Proportion of patients with targeted mutations in 2010–2023 receiving targeted therapy.

	EGFR	ALK	ROS1	BRAF	HER2	RET	MET	Total
Targeted mutation	69/636 (10.8%)	17/636 (2.7%)	3/636 (0.9%)	12/636 (1.9%)	7/636 (1.1%)	1/636 (0.16%)	14/636 (2.2%)	121/636 (19%)
Targeted therapy	48/69 (69.6%)	14/17 (82.4%)	1/3 (33.3%)	4/12 (33.3%)	3/7 (42.9%)	1 (100%)	3/14 (21.4%)	74/121 (61.2%)

EGFR: epidermal growth factor receptor; ALK: Anaplastic lymphoma kinase; ROS1: ROS proto-oncogene 1; BRAF: B-raf proto-oncogene; HER2: erb-b2 receptor tyrosine kinase; RET: RET proto-oncogene; MET: MET proto-oncogene.

**Figure 3:**
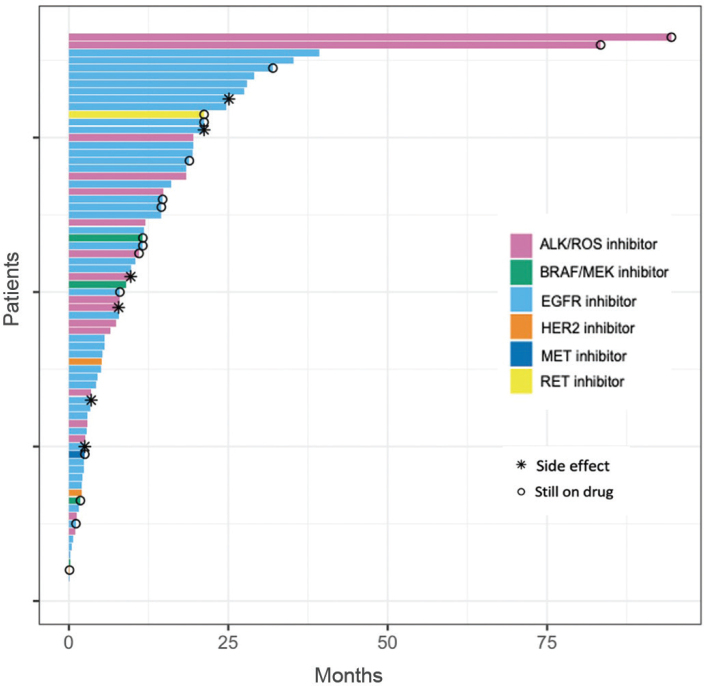
Duration of treatment in months for patients who received targeted therapy 2010–2023 (*n* = 74).

Targeted therapy uptake varied among different molecular alterations; among patients with *EGFR*-positive tumor, 69.6% (48/69) received targeted therapy, while 82.4% (14/17) of those with *ALK* translocation received therapy. These were followed by 33.3% (1/3) with *ROS1* translocation, 33.3% (4/12) with *BRAF* V600E mutation, and 42.9% (3/7) with *HER2* mutations. The only patient harboring a *RET* translocation received targeted therapy, and 21.4% (3/14) with translocation in the *MET* gene received targeted therapy. The reason that the remaining 32% did not receive targeted therapy was attributed to several factors, such as localized disease at diagnosis, selection of alternative therapy, unavailability of targeted therapy at the time, or death. Among patients who received targeted therapy, 33.8% (25/74) continued therapy for at least 1 year and 13.5% (10/74) for at least 2 years (see Figure 3). The reasons for treatment termination were disease progression, adverse events, or death.

Specifically for the last 5 years of the study period 2019–2023, where data on tumor staging are available, 200 patients were diagnosed with stage IV lung adenocarcinoma in Iceland. The proportion of patients who underwent molecular testing was 71% (142/200), 24.6% (35/142) had targetable mutations, and of those, 74.3% (26/35) received targeted therapy. Nine patients did not receive targeted therapy as they went on palliative therapy (*n* = 5), targeted therapy was not available (*n* = 2), they refused therapy (*n* = 1), or they had not progressed on current therapy (*n* = 1). Among patients harboring *EGFR* mutation, 88.9% (16/18) received targeted therapy, 75% (3/4) with *ALK* translocation, 0% (0/1) with *ROS1* translocation, 100% (2/2) with *BRAF* V600E mutation, 60% (3/5) with *HER2* mutation, 25% (1/4) with *MET* translocation, and 100% (1/1) with *RET* mutation.

## Discussion and conclusions

In this study, we show that molecular testing on lung cancer samples has increased dramatically over the past 15 years. In the last 5 years, where data are available specifically for stage IV cancers, 90% underwent molecular testing while the clinical objective is to offer comprehensive molecular analysis for all patients diagnosed with lung adenocarcinoma in Iceland. As driver mutations and gene rearrangements in certain genes can influence treatment choices in advanced lung adenocarcinoma, it is crucial that molecular testing is offered to all patients.

Targeted *EGFR* mutational analysis was first recommended in US guidelines in 2010 and in European guidelines in 2011 for never/former light smokers and tumor with non-squamous histology [12]. In 2013, an evidence-based guideline was published by the College of American Pathologists, the International Association for the Study of Lung Cancer, and the Association for Molecular Pathology recommending *EGFR* and *ALK* testing in all advanced NSCLC with an adenocarcinoma component [13]. The guidelines were updated in 2018 to include *ROS1* and *HER2*, *MET*, *BRAF*, *KRAS*, and *RET* testing in laboratories with NGS capabilities [14]. Clinical molecular testing practice in Iceland has followed and at times preceded clinical guideline recommendations.

This rapid increase in molecular testing of lung samples parallels the advancement of targeted therapy development. The driver gene most commonly mutated is *EGFR*, with around 12% of patients testing positive, which is similar to numbers from European cohorts [15]. About half of patients carry an exon 19 deletion. EGFR inhibitors were first approved for treatment of advanced disease around 2004–2005 [16, 17]. Osimertinib was approved as adjuvant therapy for limited disease in Europe in 2021 [18], and as a result, it is important to test all NSCLC cases upfront, and testing of all NSCLC adenocarcinoma cases was implemented in Iceland in year 2021. The proportion of stage IV lung adenocarcinoma samples undergoing molecular testing in Iceland between 2019 and 2023 was similar to that reported in a comparable study from the United States, where 81% of patients with metastatic NSCLC underwent *EGFR* testing [15] and higher than in a Norwegian study where > 64% underwent testing in 2017 [19].

In the last decade, several other targetable gene mutations and gene rearrangements have been identified. *ALK/ROS1* inhibitors were approved for advanced disease driven by *ALK* in the early 2010s [20] and *ROS1* rearrangements around 2016 [21]. Before 2016, testing of targetable mutations was restricted to the *EGFR* gene, and in 2016, 29.5% of lung adenocarcinoma samples, regardless of stage, underwent molecular testing. In the same year, the TST15 sequencing panel was introduced, which detects mutations in 15 genes, including *EGFR*, *KRAS*, *BRAF*, and *HER2*. In 2017, the proportion of samples submitted to sequencing analysis had gone up to 50.7%, and in 2023, 83.8% of lung adenocarcinoma samples underwent molecular testing. In the last 3–5 years, treatments for *BRAF*-driven, *HER2*-driven, *RET*, and *MET*-driven NSCLC have all been approved and adopted, further expanding the armamentarium for this cancer. During the study period, the ratio of patients receiving targeted therapies with targeted mutations has increased. Of the 49 patients diagnosed with stage IV lung adenocarcinoma in 2023, 89.8% (44/49) underwent molecular testing, 18.2% (8/44) had a targeted mutation, and of those, 75% (6/8) received targeted therapy. This is a significant increase compared to 2019, when 34 patients were diagnosed with stage IV lung adenocarcinoma, 41.2% (14/34) underwent molecular testing, 35.7% (5/14) had a targetable mutation, and 100% (5/5) received targeted therapy. A study from Norway found that > 85% of patients with EGFR mutations went on targeted therapy in 2010–2017, exhibiting a higher proportion treated [19]. In 2023, patients with stage I–IV lung adenocarcinoma were 99 in Iceland, and 83.8% (83/99) underwent molecular testing. Furthermore in our study, 34% of patients remained on therapy at 1 year [23], which is lower than in the FLAURA first-line trial studying EGFR inhibitors, where 70% remained on osimertinib and 47% remained on a comparator EGFR inhibitor at 1 year. Our study looked at multiple targeted therapies, and therefore, a direct comparison is not possible, but it is not surprising that real-world data show inferior efficacy in comparison with clinical trial data.

Ongoing research continues, and the number of potential therapeutic agents is likely to increase in the future. *KRAS* G12C inhibitors have been approved in the US, and sotorasib received conditional authorization by the EMA, but overall survival benefit is not yet clear [22], and this treatment has not been adopted in the Nordic countries.

The main strength of this study is that this is a population-based study based on comprehensive data from a nationwide cohort. Since molecular analyses are only performed centrally at Landspitali, it is unlikely that any tests performed on patients diagnosed in Iceland were missed. As targeted therapies are given orally, data from the prescription drug registry can easily be accessed and covers the whole country, including treatments prescribed in smaller hospitals.

The main weakness of the study is that there was no comprehensive staging information for the entire study period, and therefore we can only report on stage IV patients for the 2019–2023 period, which are the patients where molecular testing is critical and targeted therapy appropriately applied. Also, we did not have information on whether targeted therapy was given in the first line or subsequent lines of therapy, and we do not have information on the administration of cytotoxic therapy or immune checkpoint inhibitor therapy.

Targeted drugs were in continuous development during the study period, and new drugs became available for new targets during the period. Therefore, the treatment options are not the same each year of the study. This affected the proportion of those with a targeted mutation who received targeted therapy. Diagnostic technology also changed during the period, initially testing for single gene mutations and then moving to multigene panels. Similar developments occurred in other countries as molecular testing advanced. There are also certain limitations associated with retrospective studies as they are based on past events and older data that were not originally collected for research purposes.

In summary, we conclude that the central testing structure in Iceland has led to high NSCLC testing rates, and adaptation of new targeted therapies has been rapid.

## Supplementary Material



## Data Availability

The de-identified data that support the findings of this study are available on request from the corresponding author.
