# Supplementary material for: Targeted therapy in the treatment of lung cancer in Iceland 2010–2023
Source: Acta Oncol. 2026 Apr 9;65:45118. doi: 10.2340/ao.v65.45118 (PMC13071797; doi:10.2340/ao.v65.45118)

Supplementary material has been published as submitted. It has not been copyedited, or typeset by Acta Oncologica

Supplementary Figure 1 – The evolution of molecular testing on lung cancer at Landspitali University Hospital from 2005 to 2023.

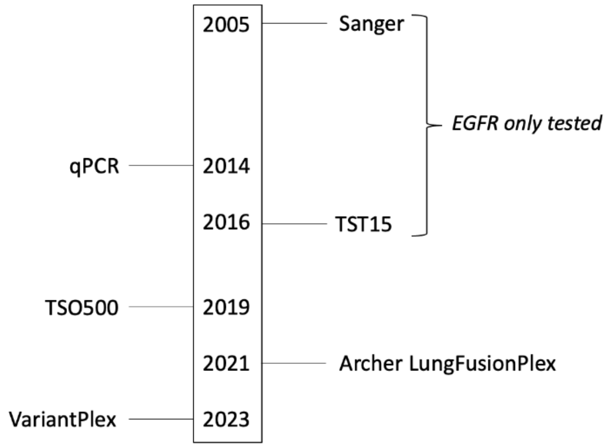

Supplementary Figure 2 – Location of mutations within the *KRAS* gene 2016-2023 (n=185) (panel A) and *EGFR* gene 2010-2023 (n=74) (panel B).

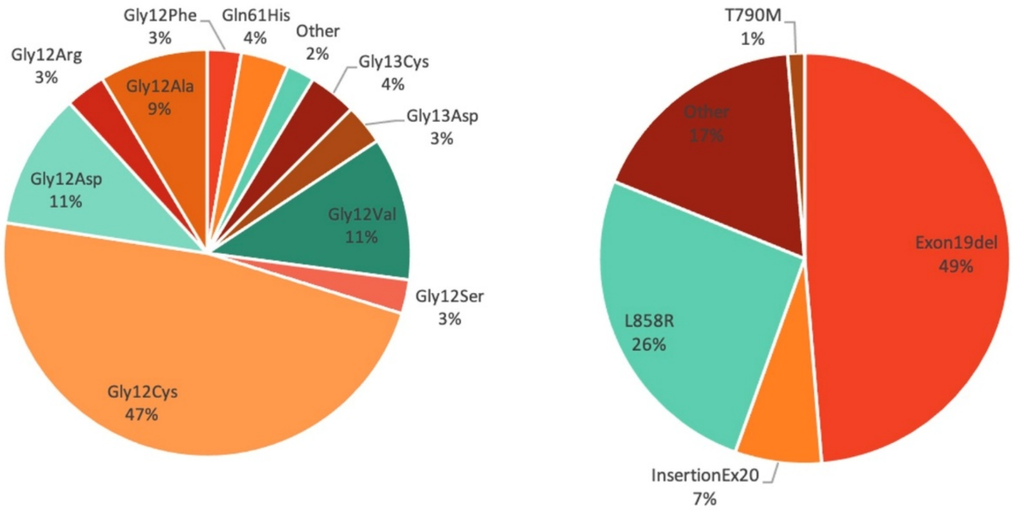

Supplement: Supplementary file 1 [file AO-65-45118-s1.pdf]
